# Inflammatory Disease of the Prostate and Its Appendages1Read at the Surgical Section. Buffalo Academy of Medicine, November 5, 1896.

**Published:** 1897-02

**Authors:** J. Henry Dowd

**Affiliations:** Buffalo, N. Y. 206 Franklin Street


### INFLAMMATORY DISEASE OF THE PROSTATE AND ITS APPENDAGES.[1]

By J. HENRY DOWD, M. D., Buffalo, N. Y.

OF ALL chronic trouble to which mankind is heir, I think, for worry, pain and inconvenience, disease of the prostate and its appendages should rank first. Bangs says, "A man with prostatitis is in a plight," and, I might add, is generally ready for a fight.

Prostatic disease in old men is readily recognised on account of the interference with urination. On the other hand, when occurring in young or middle-aged, as I will later describe, it is, in my opinion, rarely recognised, and simply because, instead of abnormal urination, the symptoms are mostly confined to the sexual function, urethral discharge and pronounced neurasthenia. The function of the prostate, as you all know, is to secrete a fluid for dilution of the semen, it also helping to fill the posterior urethra, thus causing contraction of the perineal muscles, aiding in its ready expulsion. It is slightly acid in reaction. There is one more important use of this fluid, which brings it forward as part of the sexual apparatus. The spermatozoa, until they become mixed with the prostatic fluid, are motionless. This has been demonstrated by Fuerbringer, and I have proved the above to be true many times.

The prostate, when perfectly healthy and in an adult of medium build, is about the size of a horse-chestnut, firm and tense, having two well-defined lobes, forming above, where they meet a depression. In this space internally is found tissue, rich in glands, which sometimes in advanced life undergoes pathologic changes, becoming hypertrophied, and thus causing what is called a third lobe. The structure is about evenly divided between muscular and glandular tissue.

We today recognise two varieties of prostatitis. One, where the symptoms commenced at or before forty, is gonorrheal in origin. The other, commencing at or after fifty, is an overgrowth, and is not supposed to be gonorrheal. There are also two forms. The first, a chronic inflammation of the follicles and glandular elements. The second, an overgrowth or hypertrophy of glandular and muscular tissue. I do not believe the gland is ever affected *per se*. The inflammation always, to a certain extent,

1. Read at the Surgical Section, Buffalo Academy of Medicine, November 5, 1896.

attacks the caput gallinaginis, ejaculatory ducts, seminal vesicles, or intraseminal vesicular space. Therefore I think it justifiable to follow the gynecologist in his description of inflammatory diseases of the uterus, and the like, only to say, inflammation of the prostate and its appendages.

*Etiology.*—This we must divide into two varieties,—predisposing and exciting; and I am thoroughly convinced that were not the exciting cause added, except in a limited number of cases, we should be called upon to treat but very few cases of prostatitis occurring before the forty-eighth or fiftieth year. The fire is kindled, and all that is necessary is a match to start it blazing. Or, to be more explicit, there is congestion of the gland, posterior urethra, and the like, due to early indiscretions. Later, a germ, gonococcus by name, is introduced, and upon this fertile soil inflammation with all its ravaging symptoms sets in. Cases do exist in which it is impossible to get a history of gonorrhea; and, not being a general bacteriologist, I will not venture to say the germ which is the excitant. I believe, though, it is laid at the door of the *bacillus coli communis.* The predisposing causes are many and commence at puberty. I will not take your time further than to say, with erection occurs congestion of the whole genital tract. Erection is under control of the mind, and I am sorry to say, in young boys and men, the mind is centered on the sexual apparatus a considerable of the time. Let the teacher be association or a perverted mind, the young man or boy soon learns to practise masturbation. He has a delightful feeling,—it is free from expense,—he is over-sure of not contracting disease, and it can be done in private. Therefore this life is continued for sometimes years, or until by chance he receives a book from some charlatan portraying lost manhood, insanity, and the like. He then stops this practice, pollutions occur, sometimes weekly, sometimes nightly, and even spermatorrhea develops. Now we have a good case of neurasthenia; not by a pathologic condition of the mind, but due to a more or less chronic congestion of the posterior urethra and its appendages, combined with an exalted condition of the genital nervous apparatus. He may commence a life of masturbation and be suddenly checked therein by being caught in the act, shamed or scared. Or he may by chance have the above literature thrust upon him. He discontinues the practice and seeks the company of young ladies. But every time he comes close to them, and especially do they tolerate caressing, an erection occurs, and sometimes for hours the genital organs are in a state of rigidity. Lewd pictures will excite him. In fact, a woman coasting on a bicycle will cause an erection. Thus you will see that the blood-vessels are over-distended nearly all the time, and permanent congestion must and surely does occur. To a certain extent, excess in sexual intercourse will cause the same condition of affairs.

Now comes the great question of bicycling. It is impossible to examine a prostate and say to what extent it is enlarged, unless the enlargement is quite marked, for we must take into consideration the muscular development of the person, his proportion, and the like. But, from an extensive acquaintance, nearly every one of which uses a bicycle, I have tried to formulate an opinion, not so much by examination of the glands, but by questions referable to the sexual apparatus, perineum, rectum and nervous system. My conclusions are, in a person giving no history to any extent of masturbation, never having had gonorrhea, syphilis, rheumatism or gout, and no history of injury to the perineum, from the bicycle, with a well-adjusted, open-center saddle, no permanent injury will develop. But, on the other hand, where I have found any of the above conditions present, especially gonorrhea, complicated by cystitis, posterior urethritis, deep stricture and rheumatism, I have found pronounced symptoms of neurasthenia, having their cause situated in the posterior urethra. And I have even seen cases prostrated by long rides, as the following will show :

On June 1, 1896, a wholesale liquor dealer consulted me, giving the following history : Aged 26 ; always bothered with rheumatism ; masturbated when young ; had gonorrhea fourteen months ago, apparent cure in four weeks. About six months ago, noticed a drop at meatus after urinating, looking like glycerine, and very sticky. This was not continuous, but especially after riding bicycle. Urine clean, except a few small comma-like plugs. Passes water about normally in day, and not at night ; stream is delayed in starting ; a slight dripping after completion ; no stricture. Prostate slightly enlarged and flabby ; urethra, by endoscope, nearly normal ; only one or two slightly congested spots, by use of large sound. Cold rectal psycrophore, rectal suppositories of iodide of potassium and extract belladonna, with special instructions not to ride machine over two miles a day, and then slowly. All symptoms rapidly disappeared until July 28th, when I was called to his house and found him with tenesmus of bladder at neck, urinating every ten minutes, or as soon as urine accumulated sufficiently to touch the irritated prostatic mucous membrane. Severe pains at end of micturition, and, in fact, all symptoms of acute prostatic enlargement, due to intense congestion or inflammation, but not of a severity to cause entire obstruction. The first question was, "Have you been riding lately?" "Yes. Yesterday I took a twenty-five mile ride, perspired freely, was very tired when I returned, and took two glasses of beer."

Can anyone doubt the bicycle was the cause of this condition? What do you think will be the condition of this man's prostate at 48 or 50?

I was told, the other day, of a doctor who had seen three or four cases of impotence, due to bicycle riding. I think that these cases would give a history of extensive masturbation, specific posterior urethritis, syphilis or deep stricture. Therefore it is my opinion that we should caution young or even old men against excessive use or fast riding, when a history of any of the above conditions presents itself.

The exciting cause, as I have already said, is always a germ, this being in a large number of cases the gonococcus. It is a well-known fact that in about 35 per cent. of all cases of gonorrhea the posterior urethra becomes affected, and usually about the second week. Now it does not necessarily follow that the prostate becomes involved in every case. But upon the treatment the balance hangs. The etiology of prostatic hypertrophy, where there is an overgrowth of the muscular, fibrous and glandular elements, is yet an unsettled question. In almost all cases a history of gonorrhea and stricture, with urethral instrumentation, can be elicited. Is it not possible that the patient may have had a posterior urethritis at some time, which did not present symptoms sufficient to call his attention to it, and an involvement of the gland took place early?

*Symptomatology.*—These vary according to the parts affected. First, let us take a hyperesthesia of the posterior urethra, due to masturbation. The most prominent symptoms will be nocturnal pollutions. The patient will tell you that on awaking in the morning he has headache, backache and feels languid during the day; penis is cold and shriveled. He thinks people avoid him, and he avoids them, especially the society of young ladies. He imagines everyone who looks at him knows he has practised self-abuse. He really does look anemic, hollow-eyed and sheepish. When talking to them they hang their head, as though ashamed to look you in the eye. If you look at the urine it will be found turbid, this being due to an excess of phosphates. I advise you to make no explanations, or they will soon imagine their brains are running out through their kidneys. Unless help is sought the condition becomes aggravated, diurnal pollutions set in, and occasionally true spermatorrhea. Now you have on your hands a case which would present the most aggravated symptoms of neurasthenia. The symptoms of this condition are similar to those already mentioned, only increased ten-fold. They see or feel one or several drops of whitish material several times daily, especially after defecation or the least sexual thought. They imagine people point at them on the street, describing their condition. The pains already referred to are intensely aggravated, and they imagine it is only a matter of time before they will be in a mad-house, as they are surely losing their mind. To cap the climax, they attempt sexual intercourse. It is impossible to produce an erection, or they ejaculate before entrance. They now become melancholy, and either do become an inmate of an asylum or have suicidal thoughts. Supposing they do effect an entrance and contract a gonorrhea. Almost at once the posterior urethra becomes involved with all its symptoms plus those of gonorrhea.

I consider it unnecessary to here repeat the symptoms of acute posterior urethritis. I cannot see how this condition can be mistaken or overlooked. If the perineal symptoms do not call your attention to the condition, a visual examination of the urine will certainly do so ; and an examination of the urine should be made every time you see a person, man or woman, who is suffering from urethral or bladder trouble. After the acute pain, and the like, has subsided, and there only remains the familiar drop in the morning, with tripper faden, possibly only in the first glass, emissions will be more frequent, and sometimes accompanied by a dart of pain at the end, or even the seminal discharge may be slightly bloody, showing the ejaculatory ducts have been involved. Involvement of these ducts will also cause the patient to complain of neuralgic pains in the testicles, back and groin, growing more severe as the chronicity and upward involvement take place. I might say here, that in this condition, the caput nearly always suffers. After this condition is well established, the whole life of the man seems to change. He becomes irritable to a high degree, domineering, is never satisfied with existing conditions, seeming to want to argue with everyone with whom he has any dealings. Erection can easily be produced, or as a man once described to me, " half-hearted " erections. Ejaculation takes place either before or almost immediately after entrance, and it is sometimes hours before another can be produced, when it will take a long time, and sometimes it is impossible to have an emission. If the patient is unmarried he worries as to the fulfillment of sexual calls when married ; or if married is afraid his wife may imagine him untrue. In most of the cases the pleasure is benumbed or entirely absent—especially is this the case when there is a sharp dart of pain following ejaculation. They go from one physician to another, never acknowledge any improvement and, in fact, make not only their own but the lives of everyone miserable. Sooner or later they complain of tenesmus at the end of micturition and if questioned carefully will say, " Yes, a drop or so of a something like glycerine follows the stream of urine." This is positive evidence of but one condition—involvement of the prostate gland.

Samples of urine will become both more turbid and be filled with comma-like plugs from the prostatic follicle. The stream of urine is slow in starting and followed by several drops which will be deposited generally in their trousers. Following this is the glycerine-like fluid, sometimes tinged with blood. Urination is more frequent, possibly having to get up once at night. It is not a condition of impressible urination, but rather urgent. As soon as the urine attains a certain height in the bladder, so as to allow it to enter the posterior urethra, an urgent desire at once seizes the patient. Although he can often hold off for ten or fifteen minutes, it takes concentrated thought to do so. Tenesmus is a well-marked symptom, always following urination. A fulness with shooting pains in the perineum, legs and back. When the bowels move they imagine something is slightly obstructing the passage, and this is sometimes accompanied by pain, which is very severe. In fact, one case is reported where a patient was treated for piles a year who had chronic prostatitis. Happily for the patient his condition is discovered and a treatment applied. Otherwise a condition may develop which I can best describe by the history of a case.

Aged 36, married now. Masturbated to excess when young, followed by emissions on an average of every second or third night for years. Gonorrhea in 1885, second attack in 1888 ; developed strictures which were cut by a surgeon of this city. Now complains of pains above the pubes, in back, hips and thighs, aggravated on walking, fast running or riding over rough roads. Fulness and pain in the perineum, shooting into the testicles. Severe pains on defecation. Erections cause the most excruciating pains. Urinates five or six times daily and once at night. It takes fully ten seconds for stream to start, and it is followed by dribbling and glary discharge, at one time bloody. Pain following emissions, and, in his own words, "Sexual intercourse makes me sick for a week. There is no pleasure. In fact, it is a torture, so I seldom practise it."

Examination revealed one lobe of the prostate enlarged to one-half times its fellow, which is also large. The sound No. 30 F. causes severe pain on entering the posterior urethra. This can be considered a typical picture of chronic prostatitis before the age of 36, of gonorrheal origin.

Involvement of seminal vesicles has a train of symptoms similar to those of posterior urethritis, only that the patient may at times after defecation notice a thick, tenacious discharge; this being due to hardened feces, pressing or rather stripping the enlarged pouches. These people have a history of gonorrhea. If they are married they will not be able to impregnate their wives; this being due to the accumulated pus killing the spermatozoa. At present I have a case whose right vesicle is enormously enlarged, due to a gonorrhea, who wants to know why it was before he had it he impregnated his wife, but since, nine years, has not been able to get her so, although both are perfectly healthy and he is strong and robust. I found the spermatozoa after repeated examinations to be motionless and shriveled.

They cannot tell where they feel badly, having no symptoms referable to the located disease, but are very nervous, with neuralgic pains in different parts of the body; a sudden fright makes them irritable for days; fits of melancholy supervene, when for hours or a day they will not speak unless to answer questions. After making a diagnosis of chronic vesiculitis I asked a patient for symptoms and was told, "I feel as though a veil were hanging over my face or brain. I don't seem to comprehend a situation or condition until some time has elapsed."

It is advisable in all cases where the posterior urethra has been involved in gonorrhea to examine the seminal vesicles. On the first examination it may be difficult to locate them, but with a little practice it is as easy as for the gynecologist to examine a woman and say she has a right or left enlarged tube, as the case may be.

If these conditions be due to tuberculosis the symptoms will be materially the same, excepting by digital examination, nodules will be found in the prostate, vesicles and vas deferens. And it is always well to examine the epididymis, for in a majority of cases one or both are involved.

The symptoms of chronic prostatitis commencing after the 45th year, it seems to me, are so well known that it is idle to repeat them. Suffice it to say, delay in starting the stream, dribbling afterward, having to arise to urinate at night, ammoniacal urine, exacerbations of cystitis three or four times a year, if stricture is excluded, generally point it or should cause the prostate gland to be examined.

*Treatment.*—I know of no condition in medicine or surgery which will respond more slowly to treatment than any of the conditions already described. In fact, cases at times will grow worse, even under the most appropriate remedies and applications. Each case must be studied individually and a thorough knowledge of its constitutional conditions noted. In nearly all, anemia will present itself. This should be combated with iron and tonics. Gastro-intestinal disturbances should be inquired into and treated accordingly.

Daily morning cold baths for a flabby condition of the muscular system, a change of climate and if tuberculosis is present, the best environment possible, with regular hours for sleep and meals highly nutritious, and in all conditions the cause must be found and removed.

In the first condition, nocturnal emissions, as heretofore stated, the cause is a hyperemic posterior urethra, with an exalted condition of the nervous, especially the genital nervous, apparatus.

Good advice with strict hygiene is all-important. Explain to the patient the pathological condition, that there is no trouble in the brain, that he will surely get well if persistent in treatment, and when he does recover, if unmarried, advise marriage and a strict sexual life.

Invariably emissions occur in the early morning, from 4 to 5 o'clock. The bladder is now full and as in the recumbent position there is a congested condition of the genital tract, it can easily be seen how a full bladder pressing on the vesicles will cause their overflow. Caution against drinking any fluid after 6 or 7 P. M. Evacuate the bladder before retiring, and in certain cases I advise arising at say 4 A. M. and urinating. When this has been done no emission has taken place. The bed should be moderately hard and a lateral position maintained while asleep,—this to avoid the condition already mentioned. Sometimes it may be necessary to advise a ring to be worn to prevent erections. For the patient's general condition a tonic is usually necessary. If in the urine we find phosphaturia, compound syrup of hypophosphates, containing 1.16 gr. strychnine to each dose, is admirable. In nearly all conditions it will be found necessary to give sedatives. No better drugs can be used than bromides, gelsemium and valerian morning and afternoon and a double dose at bedtime. The all-important remedy is pressure, and this can only be applied by the use of full-sized sounds, which should be passed every other day and left in for ten minutes or so. My plan is to pass a full-sized sound every other day, and on the second day to deposit a few drops of a 3 to 5 per cent. solution of silver in lanoline on the prostatic mucous membrane. In using silver in the posterior urethra, the fact must not be lost sight of that to commence with strong solutions is to do more injury than can be repaired by several treatments. Therefore, it is wise to commence applications by weak instillations or probably better still by irrigations, which will be described later. On the fourth day after removal of the sound, use the urethral psycrophore, running one or two gallons of ice water through the same. Electricity and cauterization of the ejaculatory ducts has been advised. With these measures I have had no experience.

Should specific posterior urethritis occur in connection with the before-mentioned condition, it is very advisable to cease active treatment, only combating the severe symptoms until the acute inflammation subsides. Now, with the irrigating catheter, medicate the diseased mucous membrane until the urine shows by its absence of pus shreds and comma-like plugs that only the old aggravated condition remains. If irrigation is practised every one, two or three days, according to the irritation produced, the case will be ready for its former treatment in the course of ten or twelve days.

Purposely I omitted the symptomatology of acute prostatitis. If this condition occurs during an acute or sub-acute attack of gonorrhea I do not see how it can be mistaken. The perineum close to the anus should at once be shaven and five or six leeches applied. When they fall off, the patient should be placed immediately in a sitz bath, and in the course of a few minutes the urine will flow from the urethra; or should retention not occur, the cold rectal psycrophore morning and evening will quickly reduce the swelling. Ice water must be used. To keep the good work of resolution going on, suppositories of ichthyol and belladonna should be inserted in the rectum at night. The gland will generally recover its normal size, although sometimes atrophy takes place.

The condition, early chronic prostatitis, may easily be regarded as the worst complication of gonorrhea. As already mentioned, when this condition is allowed to progress untreated there is generally but one ending—a miserable existence from about the thirty-fifth year until death closes the scene.

It is my candid opinion that when once this gland becomes invaded by a specific inflammation, unless it is recognised and treated at once, it never again becomes normal. Even though a pathologic involvement be discovered at once, time only will bring about resolution. It may be weeks, months or even a year before improvement will be manifest, and in some cases improvement never occurs.

This condition, as I have heretofore mentioned, differs from the chronic of old age and in being inflammatory; the other is hypertrophy or overgrowth. Therefore, the treatment resolves itself into one of promoting resolution and aiding absorption. The first condition is met by pressure. The Oberlander prostatic dilator is admirably adapted to this work or the largest possible sound, according to the measurement of the flaccid penile organ. But, caution: never pass a sound into the urethra while the urine contains pus or mucus visible to the naked eye! Should the meatus be small it should be divided. Should the case be recent, cold water, and by this I mean ice water, one or two gallons, should be passed through the urethral or rectal psycrophore. In a more chronic condition, water as hot as possible with the rectal instrument; but as the urethral psycrophore being metal becomes overheated this must be dispensed with. Irrigations by catheter, carried just inside the compressor muscle, give most gratifying results. Solutions of silver, 1 to 10,000, increased gradually to 1 to 300, or even 1 to 200, should be used. For absorption of the inflammatory material, I have had most excellent results with pot. iod. internally and locally, in both urethra and rectum, gr. 1½ t. i. d. I grant this will not show its work, as in a case of syphilis, but if continued its good effects will be noticed. For the rectum I use pot. iod. and belladonna, or ichthyol, in the form of suppositories, inserting one after the use of the psycrophore. In the urethra an ointment of pot. iod. and iodine in glycerine and lanoline should be deposited over the caput gallinaginis with my ointment syringe, drops ten of the above, equaling about gr. 2 pot.

iod. and iodine gr. 1-16. The openings being on the side, as the ointment is pressed from the beak it adheres to the prostatic mucous membrane. Now on withdrawal of the instrument the gland closes together, thus pressing it into the follicle, where it remains sometimes for thirty-six hours, thus exerting a local effect unattainable by any other method. Care should be taken not to lubricate the instrument with vaseline or oil, as it forms a coating to the mucous membrane, rendering the tissues impermeable. Blistering the perineum is a valuable adjunct if your patient can stand it. The general condition should receive attention, anemia, rheumatism, gout or syphilis being treated if present.

*Vesiculitis.*—Although I have not said anything as to the symptoms of the acute condition, I think rectal pain and the like, during an acute attack of gonorrhea will call your attention to an examination of these organs. Rest in bed should be insisted upon, relieving the acute pains and tenesmus by suppositories of opium and hyoscyamus, and as soon as possible use the rectal psycrophore with ice water. Should the condition become chronic the best results can be obtained by tonics, *i. e.*, syrup ferri iod. rectal psycrophore, using water as hot as can be borne every second day; and on every sixth or seventh day by stripping the vesicles according to Eugene Fuller's method. A change of climate is advocated and should be insisted upon in certain cases.

Chronic prostatitis, hypertrophy of the prostate, occurring in advanced life, when well established, seems to have but one tendency—from bad to worse until life becomes a burden. In this one condition to teach the layman antisepsis is time well spent. I acknowledge we here meet two classes of men—one that listens carefully and notes every word of advice, that appears at our office as we consider necessary, and carries out our instructions to the letter. The other will come when he has a cystitis, incontinency or distressing symptoms, and when free from the above has no use for the doctor or his advice.

About the first symptom calling our attention to prostatic trouble, providing there is no history of stricture, will be frequency of urination or an attack of cystitis. This condition is due to an irritating urine touching the oversensitive prostate or bladder mucous membrane and is caused by the bladder never thoroughly emptying itself. For, as the prostate increases in size, a pocket is formed on the posterior wall of the bladder. Sometimes diverticula exist. The bar at the neck, although in no way a part of the prostate when it is present, is an important factor to the prevention of free evacuation, thus allowing decomposition to take place in the residual urine. Certain fermentative germs acting on urea cause a formation of carbonate of ammonia and we have putrid alkaline urine. This can be obviated to a great extent by the position of the patient in passing urine and keeping the bladder free from residual urine and clean. Patients should be instructed to pass the first urine in the morning while lying upon the abdomen, slightly raising the latter to an oblique position. At least once and possibly twice a week a catheter, Nelaton's, if possible, should be passed after urination to ascertain if any residual urine remains. If any, this should be removed (up to two ounces; to go beyond this limit, although not always, is sometimes dangerous). This done, the bladder should be thoroughly washed with a sat. sol. boracic acid, taking care that no solid particles are introduced. Should we find the bladder each time to contain an extra amount of residual urine it is advisable to instruct the patient as to antisepsis and have him pass a catheter daily. For if urine is allowed to remain in the bladder, sooner or later there will be decomposition and trouble. If a mild attack of cystitis develops, nothing is better than a daily wash of arg. nit. gr. i. to aq. dist. oj., filling the bladder from the meatus by the Ultzman syringe. If there is a diminished quantity of urine secreted, hot baths and diuretics should be given, lest symptoms of uremia be developed. Patients should be instructed against getting wet, especially wet feet, sitting on the cold ground or anything cold, whereby congestion might be favored and complete retention take place. Should this occur, a rectal enemata of hot water must be given at once, followed by a sitz bath of at least fifteen minutes' duration. The urine may gradually trickle away with this procedure. If not, an attempt should be made to pass a catheter. If a possible thing avoid any metal instrument. It must be remembered the posterior urethra is distorted and more or less softened, and a false opening can easily be made. If successful in passing a catheter it may be left in twenty-four to thirty-six hours. Should you find it impossible to pass any instrument, suprapubic puncture should at once be resorted to, and here, the same as though the urine were passing through the catheter from an overdistended bladder, only a part should be removed at first sitting. The canula can be corked, and in the course of three or four hours more urine can be removed and so repeated until the viscus is empty.

Should the case come to that point where it is necessary to use the catheter every time urine accumulates sufficiently, we have to deal with the two classes already mentioned. The first class will so thoroughly carry out our instructions that they may live to a ripe old age, with little or no inconvenience excepting to have a catheter close at hand. Strict cleanliness should be insisted upon, and a lubricant of boracic acid in carbolated vaseline used for the instrument. At intervals of five to seven days they should wash the bladder with a warm antiseptic solution, never allowing more than two and one-half to three ounces to enter at once.

With the other variety of cases something more radical is called for, and for these such operations as prostatectomy, castration, ligation of the vas deferens and permanent drainage have been suggested. I will speak but very briefly about these, never having used any of them and only knowing what I have read, or seen in the hands of others.

Where a clear diagnosis can be made of an enlarged third lobe, protruding, as it does, upward at the vesicle neck, thus forming a complete dam, this can be shelled out with fair results. I have seen only one case of this kind, but I could not follow it to see results.

Castration, devised by White, of Philadelphia, is a simple operation, but the mortality is high, and although he claims 83 per cent. of his cases much relieved, in those that I have seen and from a few reported post mortems, the results are not what they should be considering the great risk to life. Ligature of the vas deferens is claimed by some to be fully as effective as castration, the results on the nervous system being less. Permanent drainage is best made by the suprapubic route.

Some time ago, in a case of prostatic hypertrophy with several tough anterior strictures, I tried to make a perineal meatus, but the wound closed against my best efforts to keep it open.

Medicine internally or locally seems to have no effect in this condition, excepting as already mentioned—namely, solutions for washing the bladder.

In conclusion, let me repeat the old familiar saying, "An ounce of prevention is worth a pound of cure." Try to prevent diseases of this gland and its appendages. Trouble commences here earlier than we have supposed. Good advice to the boys may prevent years of suffering when they are old men.

206 Franklin Street.

